# Treatment Options and Voice Outcomes for Patients With Unilateral Vocal Fold Paralysis After Thyroidectomy

**DOI:** 10.3389/fendo.2022.886924

**Published:** 2022-05-24

**Authors:** Ming-Hsun Wen, Chi-Te Wang

**Affiliations:** ^1^ Department of Otolaryngology Head and Neck Surgery, Far Eastern Memorial Hospital, Taipei, Taiwan; ^2^ Department of Electrical Engineering, Yuan Ze University, Taoyuan, Taiwan; ^3^ Department of Special Education, University of Taipei, Taipei, Taiwan

**Keywords:** vocal palsy, hoarseness, aspiration, laryngoplasty, injection

## Abstract

**Objectives:**

This study investigated the treatment options and clinical outcomes of voice therapy (VT), hyaluronic acid (HA) injection, autologous fat injection (FI), and medialization thyroplasty (MT) in patients with unilateral vocal fold paralysis (UVFP) after thyroidectomy.

**Study Design:**

Retrospective case series.

**Setting:**

A tertiary teaching hospital.

**Methods:**

This study included 51 patients with post-thyroidectomy UVFP who underwent VT (*n* = 20), HA injection (*n* = 14), FI (*n* = 12), or MT (*n* = 5) from January 2016 to June 2021. The treatment outcomes were evaluated using 10-item voice handicap index (VHI-10), maximal phonation time (MPT), and auditory perceptual rating using GRB scales (i.e., grade, roughness, and breathiness) before and 3 to 6 months after treatment.

**Results:**

Patients received HA injection presented a significantly shorter interval after thyroidectomy (mean: 4.6 months), followed by VT (6.7 months), FI (12.3 months), and MT (22.4 months). The results exhibited improvement in most of the outcomes after all of the four treatments. Additional comparisons indicated that VHI-10 scores improved the most among patients receiving MT, followed by HA, FI, and VT. The differences of MPT and GRB scores among the 4 treatment groups were non-significant.

**Conclusions:**

The results revealed that VT, HA, FI, and MT can all improve the voice outcomes of patients with post-thyroidectomy UVFP. The optimal treatment approach should be individualized according to the patient’s preference and vocal demand, and the interval between thyroidectomy and intervention.

## Introduction

Unilateral vocal fold paralysis (UVFP) is a common laryngeal disorder that may cause hoarseness, dysphagia, and even aspiration, leading to considerable impairment of quality of life (1). It commonly results from dysfunction of the recurrent laryngeal nerve (RLN) innervating the intrinsic muscles of the larynx. The etiologies of UVFP include iatrogenic, neoplastic, idiopathic, neurologic, nonsurgical trauma, and other factors (2). Iatrogenic surgical injury has become the most common cause of UVFP because of the rising number of surgeries involving RLN pathway being performed. Thyroidectomy remains the leading cause of surgery-related UVFP (2, 3).

A systematic review of 27 articles and 25,000 patients revealed that the average incidence rates of temporary and permanent UVFP after thyroidectomy are 9.8% and 2.3%, respectively (4). In a large multi-institutional study, RLN injury occurred in nearly 6% of thyroid surgeries (5). The incidence of RLN palsy after thyroid surgery is up to 8% for transient palsy and ranges from 0.3% to 3% for permanent palsy (6–8).

Multiple treatment modalities have been proposed for UVFP. For example, voice therapy (VT) may be applied as a conservative and noninvasive treatment with documented clinical effectiveness (9). Injection laryngoplasty (IL), which was first introduced by Dr. Bruening in 1911, is another common treatment option for UVFP (10). Owing to advances in endoscopic technology, various injection approaches may be used in the treatment of UVFP according to the patient’s tolerance and the surgeon’s preference. Depending on the longevity of the injection materials used, IL can be performed as a temporary or long-lasting means of improving glottal insufficiency. The list of injection materials may include collagen, hyaluronic acid (HA), calcium hydroxylapatite, and other synthetic materials (11, 12). Autologous fat injection (FI) is another well-established procedure for UVFP. Lipoinjection requires overinjection by some degree to correct for the expected resorption of fat in the first 4 to 6 weeks after the procedure (13).

In contrast to IL, of which the sustainability depends on the materials used, medialization thyroplasty (MT) is considered a definite long-term treatment for UVFP. MT is performed by creating a window in the thyroid cartilage and pushing the paralyzed vocal fold medially by an implant, such as a silicone block, Gore-Tex strip, or titanium plate (14–16). Similar to IL, MT is mostly performed under local anesthesia to facilitate real-time audio feedback. It can be performed in conjunction with arytenoid adduction to further improve posterior glottal closure (17).

Although some studies have compared the surgical outcomes of patients with UVFP (18), few have focused specifically on the management and effectiveness of post-thyroidectomy UVFP. Therefore, this study investigated the voice outcomes associated with different treatment options for patients with UVFP after thyroid surgery. Our research goal was to elucidate the effectiveness of clinical treatment options for this specific clinical scenario.

## Materials and Methods

### Study Setting

**Figure 1:**
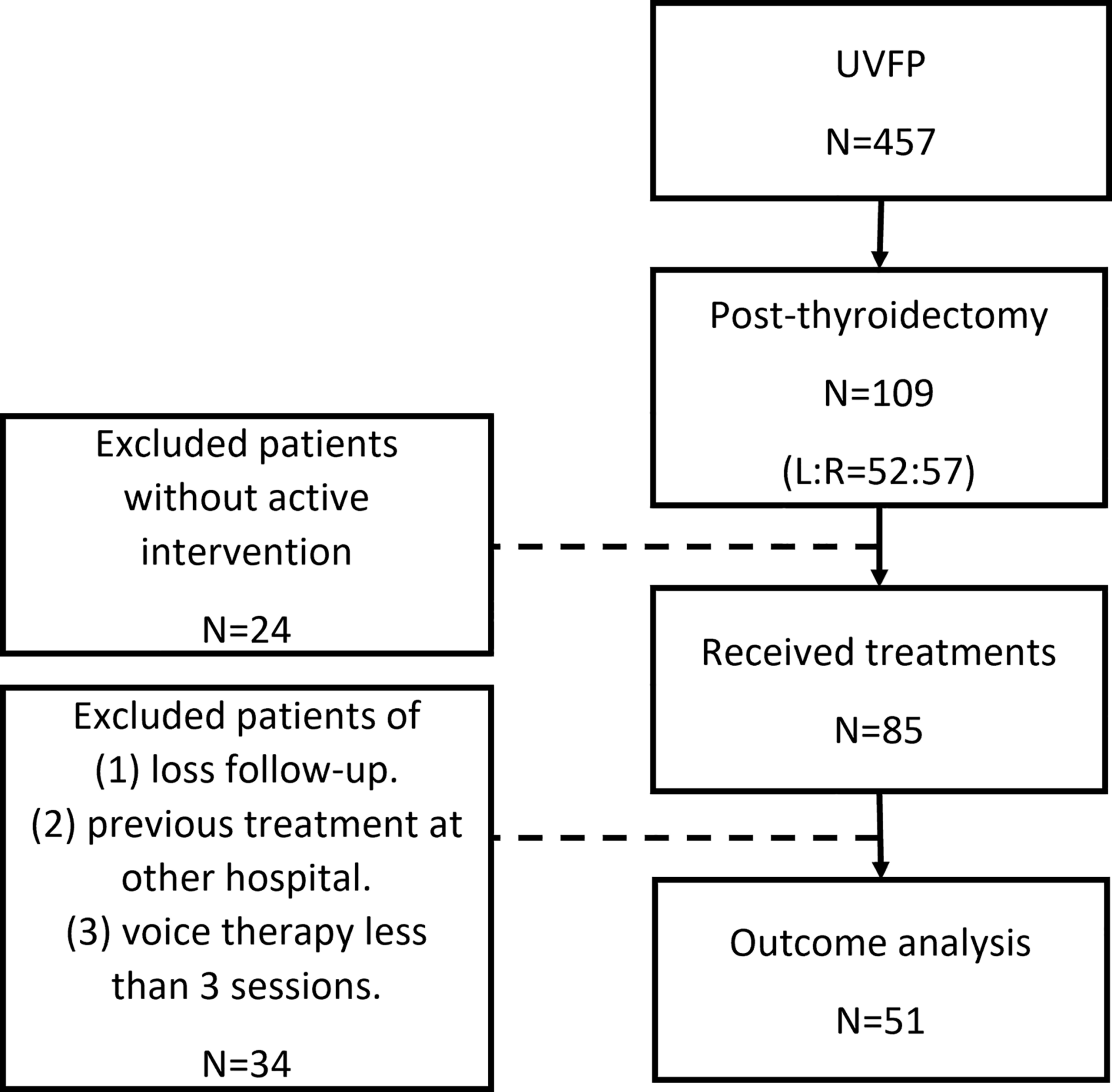
Flow chart of study cohort.

We retrospectively reviewed the medical charts of patients who were diagnosed with UVFP after thyroidectomy and visited the voice clinic of a tertiary teaching hospital from January 2016 to June 2021. UVFP was clinically diagnosed on the basis of an immobile vocal fold with an atrophic, bowing appearance. Patients who did not receive active intervention, who received preceding treatments at another hospital, who were lost to follow-up or who received voice therapy less than three sessions were excluded (**Figure 1**). This study protocol was approved by the Research Ethics Review Committee of Far Eastern Memorial Hospital (FEMH No. 111032-E).

### Procedures

VT sessions were conducted by a senior therapist with more than 10 years of clinical experience. All the participants were scheduled for one session of individual VT per week and were instructed to perform further practice at home after each session. The treatment strategies, which included vocal function exercise, pushing exercise, water resistant therapy, and hard glottal attacks (19, 20), were tailored according to clinical judgement. To ensure a sufficient treatment effect, patients who attended fewer than three sessions of VT were excluded from the study.

**Figure 2:**
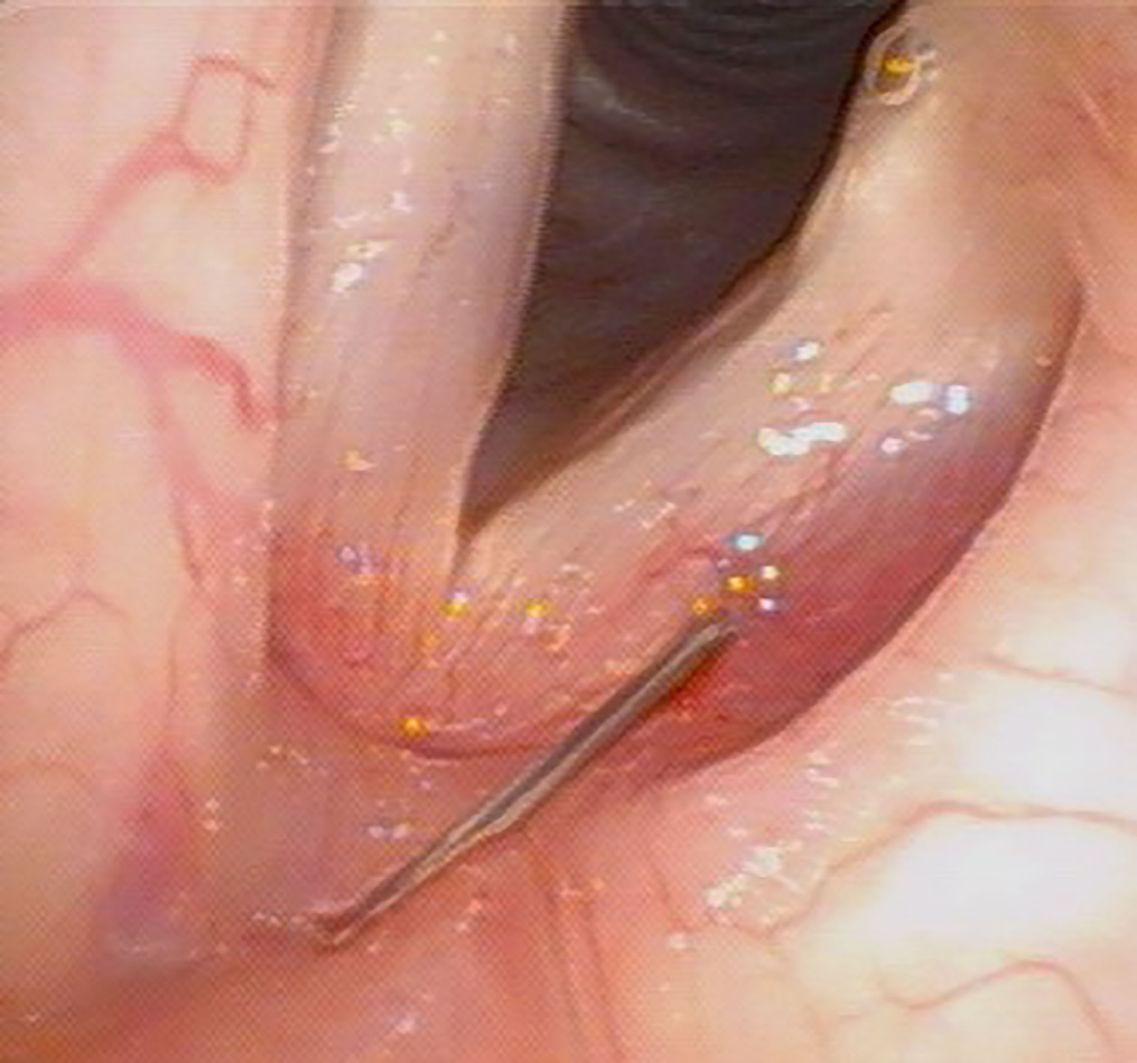
A 40-year-old woman with left UVFP received HA injection *via* transcutaneous approach under local anesthesia 3 months after thyroidectomy.

Vocal fold HA injection was conducted under local anesthesia in an office setting. A 10% lidocaine solution was sprayed on the pharynx, tonsils, vallecula, and epiglottis, followed by laryngeal gargling of a 2% lidocaine solution (21). We injected HA into the vocal folds *via* a transcutaneous or a transoral approach (22), and the injection locations were (1) lateral to the vocal process and (2) the middle third of the vocal fold at the depth of the vocalis muscle (**Figure 2**). The required amount of augmentation was determined according to acoustic feedback and usually ranged from 0.5 to 1.0 mL. The entire procedure was completed within 15 minutes in cooperative patients.

**Figure 3:**
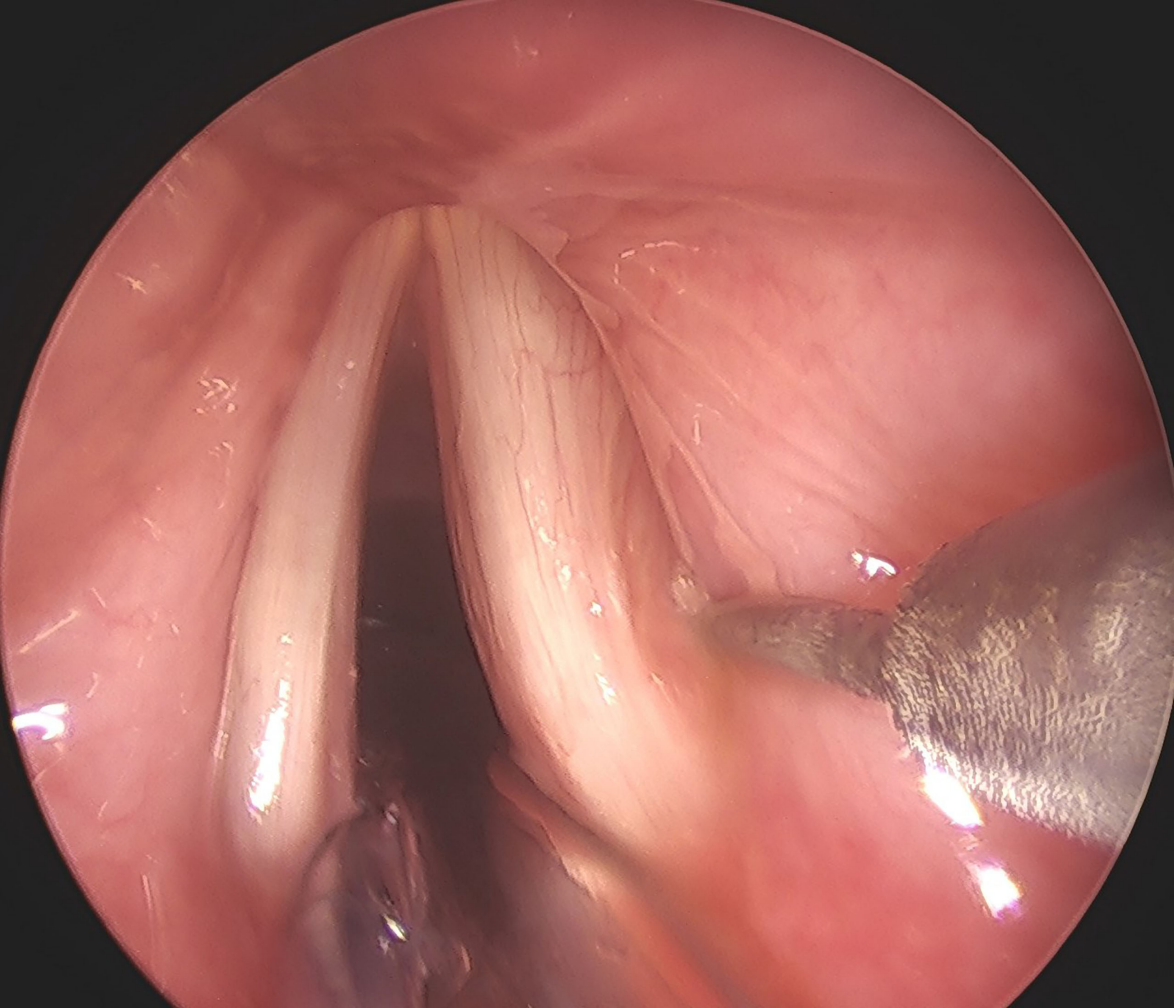
A 56-year-old woman with right UVFP received FI under general anesthesia 9 months after thyroidectomy.

We performed FI under general anesthesia. Autologous fat was obtained from the lower abdomen through liposuction *via* a small peri-umbilical incision. After infiltrating tumescent solution, we aspirated 10 to 20  mL of subcutaneous adipose tissue. We further rinsed the fat tissue with normal saline to remove blood clots, and the purified fat globules were loaded into a 1-cc insulin syringe. The autologous fat was then injected into vocal folds under the guidance of a direct suspension laryngoscope (**Figure 3**), and the target area was the posterior third of the vocalis muscle, just lateral to the vocal process. To compensate for fat loss or reabsorption, we generally overinjected 20% to 30% of the fat (23).

**Figure 4:**
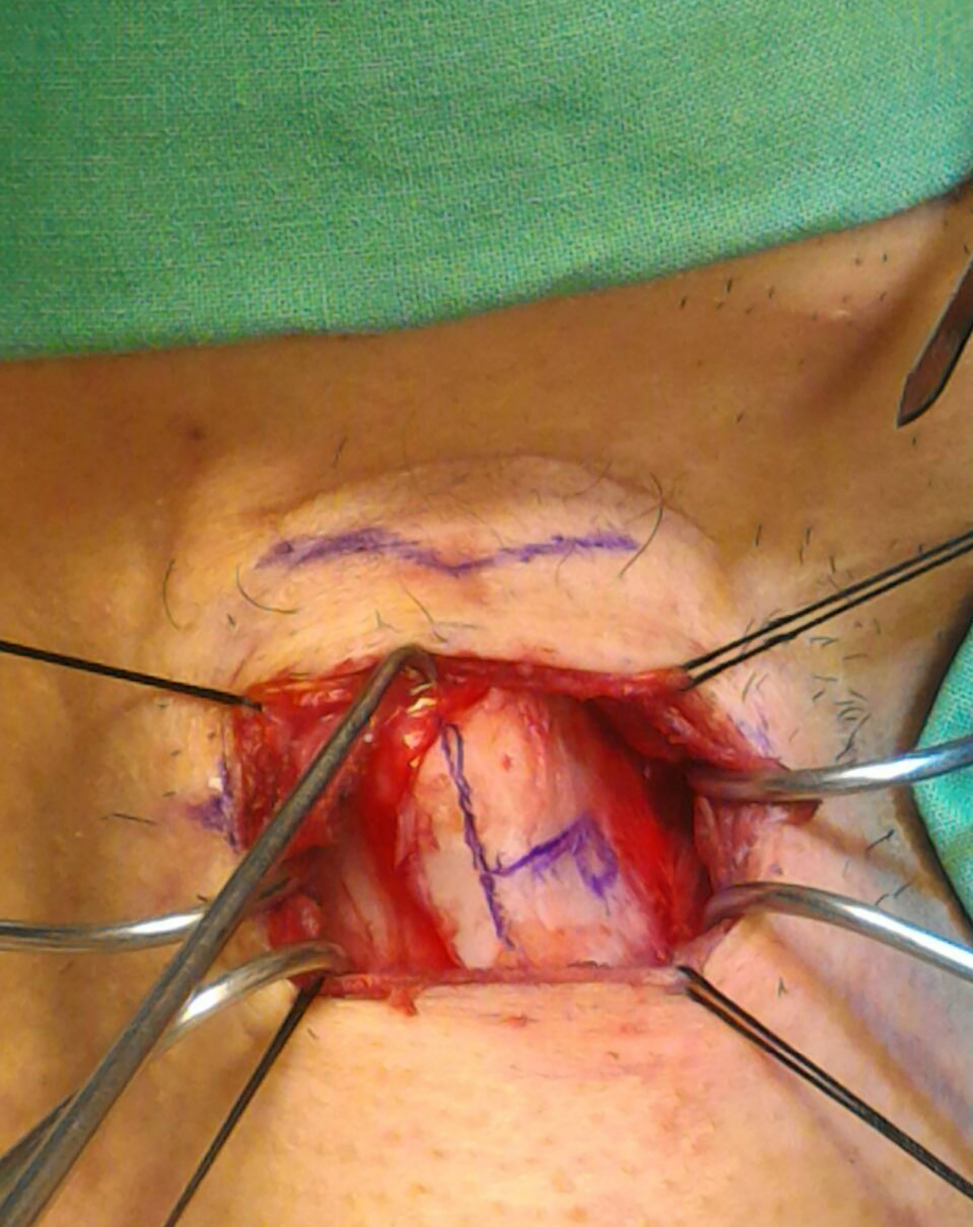
Measuring the thyrotomy window during MT for left UVFP.

All the MT procedures were performed under local anesthesia combined with light intravenous sedation. A horizontal skin incision was made at the level of the mid-height of the thyroid cartilage. After we elevated the platysma and retracted the strap muscle, the thyroid cartilage was exposed. The thyrotomy window was created 3–4 mm above the lower margin of the thyroid cartilage, 6–8 mm lateral to the midline of the thyroid cartilage (**Figure 4**). The window was approximately 3–4 mm high and 5–8 mm wide depending on the size of the thyroid cartilage (24). We inserted a Gore-Tex sheet to medialize the paralytic vocal fold, and adjusted the depth of medialization according to auditory feedback by asking the patient to phonate intra-operatively. Once the voice quality was tuned, the implant was sutured to the thyrotomy window and closed using a small piece of bone wax. After careful hemostasis, the wound was closed in layers without the need to place a drain.

### Outcomes and Statistics

Treatment outcomes were investigated using the following parameters: (1) the 10-item Voice Handicap Index (VHI-10), (2) Maximum Phonation Time (MPT), and (3) perceptual rating of voice quality using GRB (Grade, Roughness, Breathiness) scales (rated 0=normal, 1=mildly deviated, 2=moderately deviated, or 3=severely deviated. Post-operative outcome were measured between 3-6 months after the treatments. One-way ANOVA and Chi-square test were used to compare the difference between the four treatment groups. Treatment outcomes of various modalities was analyzed by Generalized Estimating Equations (GEE). P<0.05 was considered statistically significant.

## Results

### Population

We screened 457 patients with UVFP and identified 109 patients who developed UVFP after thyroidectomy. The right and left vocal folds were involved in 57 and 52 cases, respectively. We excluded 24 patients who did not receive active intervention and 34 patients who were lost to follow-up, who were already treated at another hospital, or who had received fewer than three sessions of VT. Ultimately, 51 patients were included in subsequent analyses. (**Figure 1**).

Of the 51 patients, 20, 14, 12, and 5 received VT, HA, FI, and MT, respectively. The age range was 20 to 81 years, without significant differences among the treatment groups. Other demographics, namely sex, smoking, and alcohol consumption were also not significantly different among four groups (**Table 1**). The time interval between thyroidectomy and intervention for UVFP was significantly shorter in patients receiving HA and VT, compared with patients receiving MT and FI (p<0.01, ANOVA). The average interval between VT and subsequent treatment modalities was 3.4 months (standard deviation 0.8 months), corresponding to a mean of 9.5 VT sessions (standard deviation: 4 sessions).

**Table 1:** Characteristics of the 51 patients with post-thyroidectomy UVFP receiving various treatments.

	VT (N=20)	HA (N=14)	FI (N=12)	MT (N=5)	p-value
**Age**	57.1 ± 10.2	51.5 ± 18.7	57.5 ± 12.8	51.4 ± 15.1	0.61*
**Sex**					
Female/Male	18/2	11/3	10/2	4/1	0.82^#^
**Smoking**					
Active/Others	0/20	0/14	0/12	0/5	–
**Alcohol**					
Active/Others	0/20	1/13	0/12	1/4	0.16^#^
**Time Interval (Month)**	6.7 ± 6.7	4.6 ± 2.3	12.3 ± 8.1	22.1 ± 14.4	<0.01*

*: One-way ANOVA.

^#^: Chi Square Test.

### Treatment Outcomes

Almost all treatment outcome parameters improved significantly after treatment in all the four groups (**Table 2**). Improvement of MPT in the FI group and GRB scores in the MT group exhibited borderline significance.

**Table 2:** Voice outcomes of the 4 different treatment groups.

Parameters	VT (N=20)		p-value^+^	HA (N=14)		p-value^+^	FI (N=12)		p-value^+^	MT (N=5)		p-value^+^
	Pre	Post		Pre	Post		Pre	Post		Pre	Post	
	Mean ± SD	Mean ± SD		Mean ± SD	Mean ± SD		Mean ± SD	Mean ± SD		Mean ± SD	Mean ± SD
**VHI-10**	30.8 ± 6.1	22.9 ± 9.2	<0.01	33.2 ± 4.4	14.1 ± 11.0	<0.01	30.9 ± 5.9	16.3 ± 7.2	<0.01	30.8 ± 13.7	7.6 ± 4.6	0.04
**MPT**	5.0 ± 2.6	6.8 ± 3.5	0.03	4.2 ± 2.3	9.4 ± 5.5	<0.01	4.3 ± 1.7	8.3 ± 7.0	0.06	6.0 ± 4.3	10.3 ± 4.9	0.05
**GRB(sum)**	5.2 ± 1.8	3.9 ± 1.3	<0.01	6.0 ± 0.9	2.9 ± 2.2	<0.01	5.2 ± 1.3	3.1 ± 1.9	<0.01	5.4 ± 1.5	2.2 ± 2.3	0.08

^+^: Paired t test.

**Figure 5:**
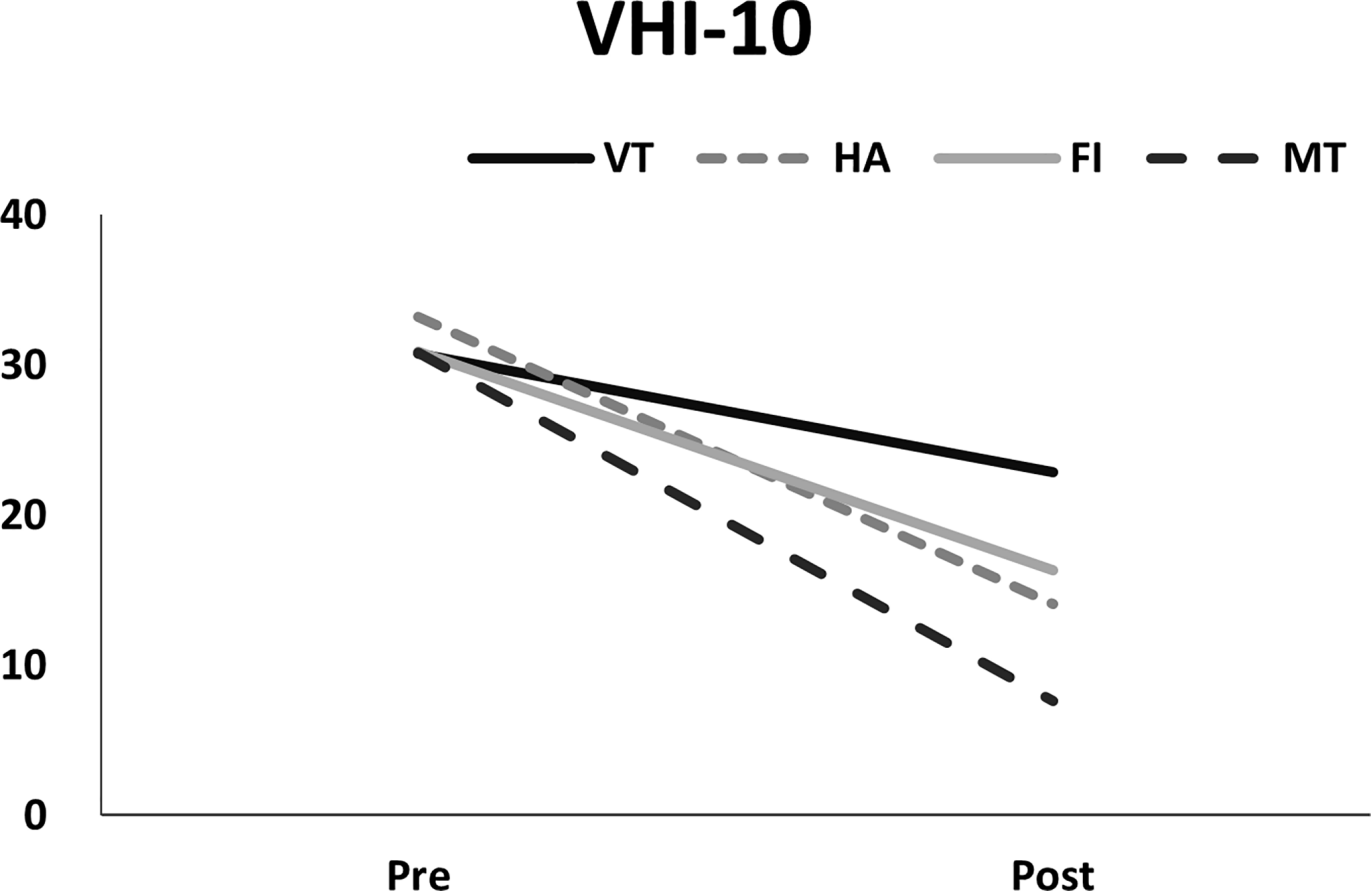
Pre- and post-measurements of VHI-10 in different treatment groups.

**Figure 6:**
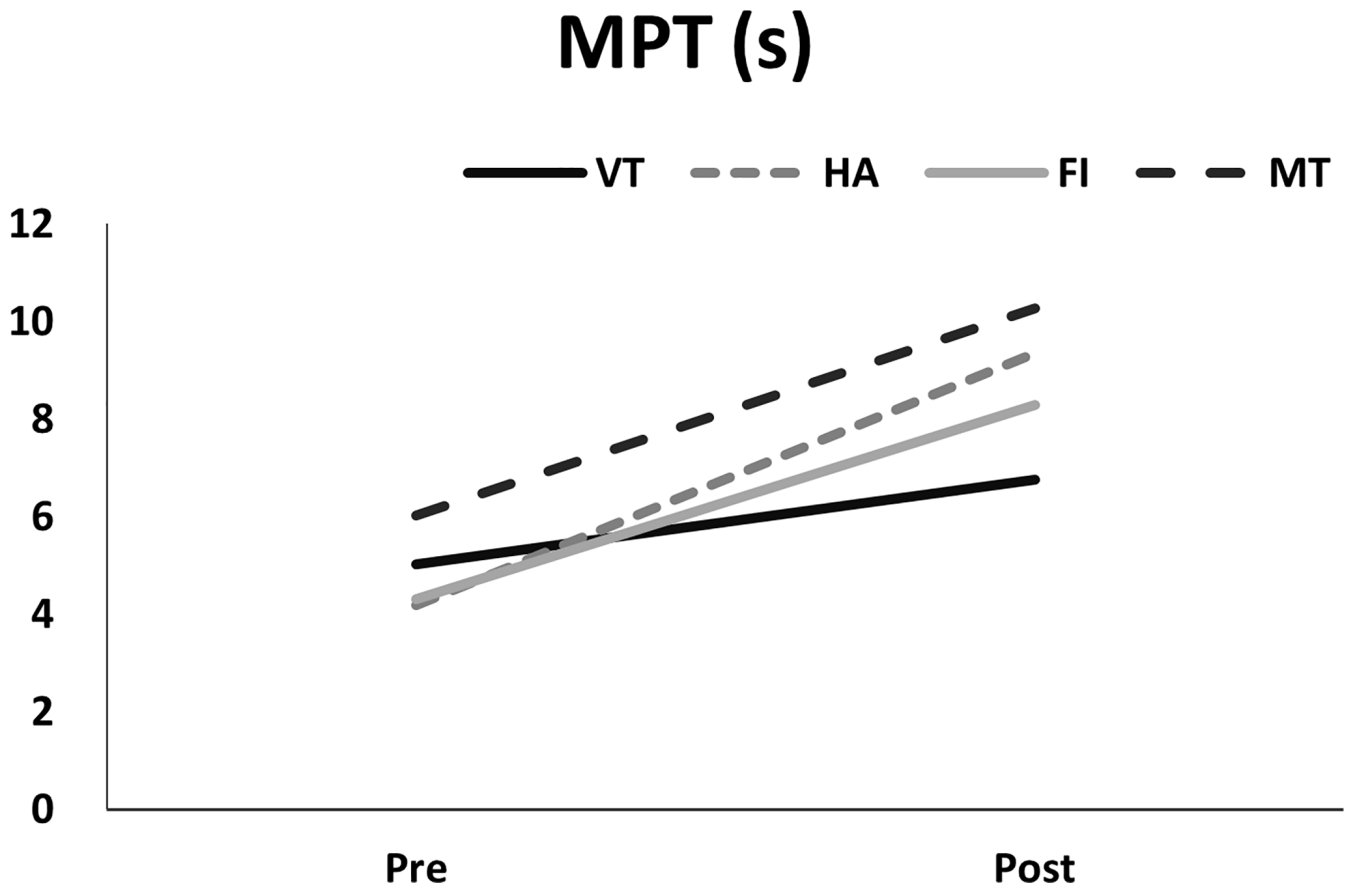
Pre- and post-measurements of MPT (seconds) in different treatment groups.

**Figure 7:**
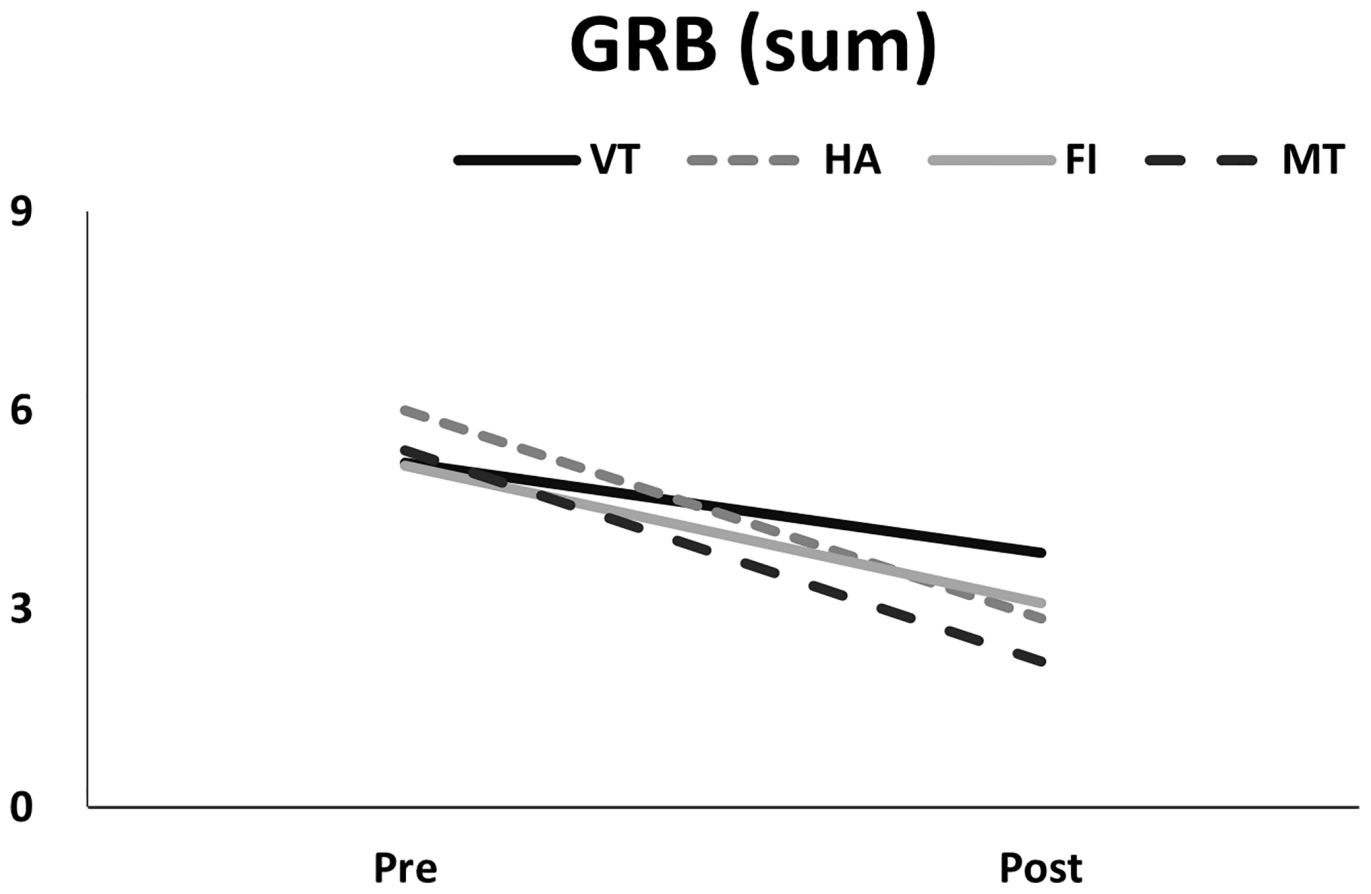
Pre- and post-measurements of the summation of GRB scores in different treatment groups.

Examining the VHI-10 scores before and after interventions, the MT group exhibited the greatest improvement (**Figure 5**, p<0.01, GEE), followed by the HA, FI, and VT groups. Regarding MPT, generalized estimating equation analysis revealed significant improvement after treatment (**Figure 6**, p<0.01), but no differences were identified among the treatment groups (p=0.59). Similarly, the patients’ GRB scores all improved significantly after treatment (**Figure 7**, p<0.01), but no significant differences were identified among the treatment groups (p=0.56).

## Discussion

Treatment for UVFP after thyroidectomy is a perennial challenge. In fact, RLN injury after thyroidectomy is a common allegation in malpractice litigations (25). According to our clinical experience, timely intervention can substantially attenuate the negative impact of UVFP after thyroidectomy and reduce patients’ willingness to file law suits. In the present study, all the treatment modalities resulted in significant voice improvement in patients with UVFP after thyroidectomy, which is consistent with the conclusions of a systematic review (18). Nevertheless, the optimal treatment strategy should be individualized according to the time interval after thyroidectomy, vocal demand, severity of complications, compliance, and expectations.

During thyroid surgery, the RLN may undergo stretch injury or accidental transection (26). In clinical practice, when vocal palsy is identified after surgery, thorough and regular follow-up every 3 to 4 weeks until the patient recovers is recommended. For patients with high vocal demand and strong motivation, VT can be applied as the first-line treatment (27). Voice training minimizes the impact of UVFP on daily life and helps patients endure the recovery period. Previous studies had documented that early referral often leads to a superior VT outcome (28). The treatment methodologies employed (e.g., the glissando maneuver to activate the cricothyroid muscles, hard glottal attacks to strengthen the adductor muscles, and resonant VT) may vary among speech pathologists. Most of these approaches are intended to enhance the laryngeal compensatory mechanisms from the healthy side. Our study results also indicated that although the degree of voice improvement associated with VT was slightly lower than that associated with the other surgical modalities, VT is still effective and well tolerated. VT remains the most common treatment for post-thyroidectomy UVFP in our routine practice (**Table 1**).

For patients who failed to improve after VT or presented with active dysphagia shortly after thyroidectomy, IL is the most practical choice for temporary intervention. Our practice mostly uses HA in IL procedures because of its high tissue compatibility and easy access. Our previous study showed that symptomatic relief could last for 9–14 months after HA injection (22), indicating that HA is a suitable material for temporary correction of UVFP (29). Early HA injection might promote a more proper vocal fold position with improved outcomes (30), which explains why only a small fraction of the patients in the present study underwent definite MT. The greatest advantage of HA injection is that it can be performed in the clinic under local anesthesia, meaning patients do not require an additional procedure in the operating room to correct complications from the preceding thyroidectomy. HA injection can also provide rapid (sometimes immediate) voice recovery, which is beneficial for patients with high vocal demand. In addition, early HA injection can reduce the anxiety of uncertain voice recovery and ease the tension between the thyroid surgeon and the patient.

When functional recovery of phonation and swallowing is absent after 9-12 months of observation, treatment modalities with longer-lasting effects (e.g., FI and MT) are indicated. Despite the wide acceptability and tissue compatibility of autologous fat, clinical practitioners must account for the uncertain survival rates of fat grafts. Studies have reported high failure rates following FI, and patients may require revision surgery (31, 32). The variability in the clinical outcomes of the procedure can be explained by the different techniques for fat harvesting and injection. In the present study, the effects of IL with HA and fat remained similar for up to 6 months. Limited by the lack of long-term follow-up records, we are unable to determine whether fat may sustain as a long-term filler, as suggested in other studies (33).

MT is widely applied as a permanent treatment for patients with UVFP and provide satisfactory outcomes on experienced hands. However, performing an additional open surgery to correct vocal palsy caused by a previous thyroidectomy can induce substantial psychological stress for the patient. In the present study, only five of the patients agreed to undergo MT after thyroidectomy. Nevertheless, our results indicated that the voice outcomes associated with MT were superior to the other treatment modalities, especially in terms of patient-reported VHI-10 scores. A likely explanation for this trend is that MT is performed under local anesthesia, thereby enabling the patient to actively participate in the vocal tuning process.

Our study has several limitations. First, the treatment modalities were not randomized but based on shared decision between the otolaryngologist and the patient. Second, the short follow-up period limits the interpretation for long-term treatment outcomes. In addition, the treatment results of UVFP strongly depend on the surgeon’s experience, and outcomes of VT may also be influenced by the patient’s motivation and active adherence (34). Further studies with longer follow-up periods and larger samples are warranted to evaluate and compare the long-term effects of different treatment modalities for thyroidectomy-related UVFP.

## Conclusion

UVFP is common after thyroidectomy, and common treatments include VT, HA, FI, and MT. Our results indicated significant improvements of voice outcomes associated with all these treatments. Clinical decisions may be tailored according to the surgeon’s experience, the patient’s preference and vocal demand, and the interval between thyroidectomy and intervention.

## Data Availability Statement

The raw data supporting the conclusions of this article will be made available by the authors, without undue reservation.

## Ethics Statement

The studies involving human participants were reviewed and approved by Research Ethics Review Committee of Far Eastern Memorial Hospital (FEMH No. 111032-E). Written informed consent for participation was not required for this study in accordance with the national legislation and the institutional requirements.

## Author Contributions

M-HW: Manuscript preparing and drafting, data analysis, C-TW: Study conceptualization, data analysis, manuscript drafting and proof reading. All authors contributed to the article and approved the submitted version.

## Funding

This work was supported by grants from the Far Eastern Memorial Hospital (FEMH-2022-C-067 and FEMH-2022-C-068).

## Conflict of Interest

The authors declare that the research was conducted in the absence of any commercial or financial relationships that could be construed as a potential conflict of interest.

## Publisher’s Note

All claims expressed in this article are solely those of the authors and do not necessarily represent those of their affiliated organizations, or those of the publisher, the editors and the reviewers. Any product that may be evaluated in this article, or claim that may be made by its manufacturer, is not guaranteed or endorsed by the publisher.

## Abbreviations

VT, Voice therapy; HA, Hyaluronic acid; FI, Fat injection; MT, Medialization thyroplasty; UVFP, Unilateral vocal fold paralysis; VHI-10, 10-item Voice Handicap Index; MPT, Maximal phonation time; GRB, Grade, Roughness, Breathiness; RLN, Recurrent laryngeal nerve; GEE, Generalized estimating equation.
